# Few-shot prediction of amyloid β accumulation from mainly unpaired data on biomarker candidates

**DOI:** 10.1038/s41540-023-00321-5

**Published:** 2023-11-23

**Authors:** Yuichiro Yada, Honda Naoki

**Affiliations:** 1 Laboratory of Data-driven Biology, Graduate School of Integrated Sciences for Life, Hiroshima University, Kagamiyama, Higashi-hiroshima, Hiroshima, 739-8526 Japan; 2 Kansei-Brain Informatics Group, Center for Brain, Mind and Kansei Sciences Research (BMK Center), Hiroshima University, Kasumi, Minami-ku, Hiroshima, 734-8551 Japan; 3 Laboratory of Theoretical Biology, Graduate School of Biostudies, Kyoto University, Yoshidakonoecho, Sakyo, Kyoto, 606-8315 Japan; 4 Theoretical Biology Research Group, Exploratory Research Center on Life and Living Systems (ExCELLS), National Institutes of Natural Sciences, Okazaki, Aichi 444-8787 Japan

**Keywords:** Stochastic modelling, Bayesian inference, Biomarkers

## Abstract

The pair-wise observation of the input and target values obtained from the same sample is mandatory in any prediction problem. In the biomarker discovery of Alzheimer’s disease (AD), however, obtaining such paired data is laborious and often avoided. Accumulation of amyloid-beta (Aβ) in the brain precedes neurodegeneration in AD, and the quantitative accumulation level may reflect disease progression in the very early phase. Nevertheless, the direct observation of Aβ is rarely paired with the observation of other biomarker candidates. To this end, we established a method that quantitatively predicts Aβ accumulation from biomarker candidates by integrating the mostly unpaired observations via a few-shot learning approach. When applied to 5xFAD mouse behavioral data, the proposed method predicted the accumulation level that conformed to the observed amount of Aβ in the samples with paired data. The results suggest that the proposed model can contribute to discovering Aβ predictability-based biomarkers.

## Introduction

Prediction is a powerful approach to evaluating the association between the input and target values. To predict a target value based on an individual input value, it is usually necessary to acquire paired data consisting of input and target values obtained from the same individual. However, in biomedical research, especially in the field of neurodegenerative diseases, obtaining such paired data in different modalities is laborious and often avoided. The same holds true for the research field of biomarker discovery in Alzheimer’s disease (AD), the most common cause of dementia^1,2^.

AD is a neurodegenerative disease in which neurons in the brain gradually die, causing progressive cognitive decline characterized by memory loss, impaired judgment and reasoning skills, communication difficulties, and changes in personality and behavior. Most cases of AD progress sporadically, and many patients have no family history of AD^3^. The genetic background of sporadic AD has been extensively investigated, revealing the complex genetic architecture of late-onset neurodegenerative disease^4–7^. The diagnosis or risk foresight of AD before the onset of the irreversible progression of neuronal loss may enable the potential treatment of the disease or the administration of appropriate symptomatic medication. The rapidly growing population of affected people raises an urgent demand for developing novel biomarkers and prediction methods for AD before irreversible neurodegeneration^8–13^.

**Fig. 1:**
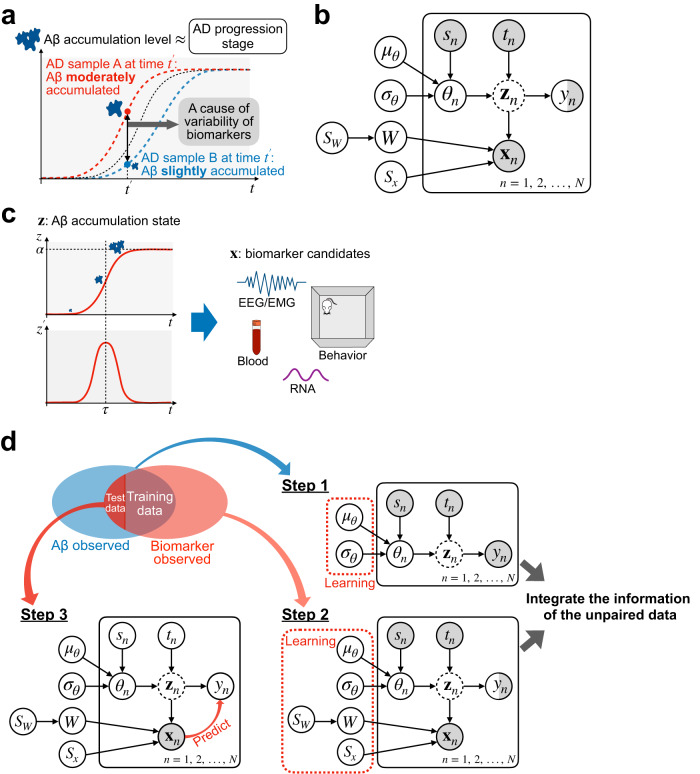
Concept and schematic representation of the proposed model. **a** Alzheimer’s disease (AD) model samples (sample A and B) at the same age may have considerably different degrees of amyloid-beta (Aβ) accumulation, which may correlate with the progression stage of very early AD. **b** The graphical model representation of our proposed model. **c** The concept of our model. Aβ accumulates in a particular brain region according to a logistic function over time. Observed data on biomarker candidates are generated depending on the accumulation level and the instantaneous accumulation speed of Aβ in the brain. **d** Learning and prediction steps of the proposed model. The model learns the parameters that integrate mostly unpaired data and predicts the quantitative accumulation level of Aβ based on observed data. In the first step, the distributions of hyper-parameters are inferred from the observed Aβ value. In the second step, the distributions of all parameters and hyper-parameters were updated using the observed biomarker candidate data. Third, the quantitative predictability of Aβ accumulation based on observed biomarker candidates was evaluated.

In AD, the gradual accumulation of amyloid-beta (Aβ) precedes irreversible neurodegeneration^1,2,14^. The Aβ accumulation level, therefore, can be an indicator of disease progression in the very early phase^15–17^. The indirect assessment of Aβ, Aβ imaging in the brain using positron emission tomography (PET), and the assessment of Aβ in cerebrospinal fluid (CSF) potentially facilitate AD onset prediction^1,2,18–20^. Recently, phosphorylated plasma tau was reported as a promising blood biomarker candidate to detect the accumulation status of Aβ in a human cohort^8,21^. However, the current biomarker candidates in AD are not pair observed with the direct quantification of Aβ; thus, the effectiveness of the candidates in the very early phase is unknown. During biomarker discovery, researchers usually evaluate the binary predictability, i.e., healthy/disease, mild cognitive impairment/AD, amyloid positive/negative, or significant difference between groups using statistical tests. However, the progression timing and disease dynamics may be heterogeneous even within each group. Especially during the accumulation process, such heterogeneity could mask the differences researchers aim to detect (Fig. 1a). The direct quantitative observation of Aβ can be performed only in the brain tissue of humans after death or animals after euthanization. Owing to the difficulty of paired observations, the quantitative accumulation level of Aβ in the brains of individuals, which may reflect the progression state of early AD pathology, may have been overlooked in past biomarker discoveries.

In the present study, to overcome this problem, we developed a hierarchical Bayesian model that describes the Aβ accumulation process and observation of biomarker candidates utilizing mostly unpaired data (Fig. 1b). In the model, Aβ deposits over time according to a logistic function, whose parameters are unique to each sample. The effectiveness of the biomarker candidates can be evaluated by the predictability of the quantitative accumulation level of Aβ. Owing to the Bayesian probabilistic formulation, the model can naturally integrate mostly unpaired data through few-shot learning, increasing the predictability of accumulation levels based on the observed biomarkers. By applying the model to the behavioral data sets of 5xFAD mice^22^, we predicted the accumulation level of Aβ solely from behavioral data from mostly unpaired data, supporting the concept of biomarker discovery based on predictability.

## Results

### Hierarchical Bayesian model of Aß accumulation

To represent the pathogenesis of AD, we developed a mathematical model describing the accumulation process of Aβ in the brain and the observation process of Aβ and biomarker candidates. Here, we assumed an AD study using model animals, and each sample was identified as belonging to either healthy wild type (WT) or AD model animals. In the model, Aβ accumulates over time according to the logistic function (Fig. 1c):

1 $${z}_{n}=\frac{{\alpha }_{n}}{1+\exp \left\{-{\beta }_{n}\left({t}_{n}-{\tau }_{n}\right)\right\}},$$

where the suffix $n\in \{1,2,\ldots ,{N}\}$ represents the index of the sample, and ${t}_{n}$ is the age of the month in which the observation of sample $n$ was conducted. ${\theta }_{n}=\left\{{\alpha }_{n},{\beta }_{n},{\tau }_{n}\right\}$ is a set of parameters depending on the individual animals and ${\alpha }_{n}$, ${\beta }_{n}$, and ${\tau }_{n}$ denote the maximum level of Aβ accumulation, steepness of Aβ accumulation, and critical period to reach half of the maximum Aβ, respectively. To express heterogeneity in the Aβ accumulation process among animals, different values of ${\theta }_{n}=\left\{{\alpha }_{n},{\beta }_{n},{\tau }_{n}\right\}$ are assigned to the individual animals, following the distribution shared within the same type of animals ${s}_{n}$, i.e., WT or AD model animals.

The observed amount of Aβ, ${y}_{n}$, is subject to noise as ${y}_{n}={z}_{n}+{\sigma }_{y}{\xi }_{n}$, where ${\sigma }_{y}$ and ${\xi }_{n}$ indicate the noise strength and Gaussian noise with zero mean and unit variance, respectively. The observed data on biomarker candidates ${{\bf{x}}}_{n}\in {{\mathbb{R}}}^{L}$ were assumed to reflect ${z}_{n}$ and its temporal derivative ${{z\text{'}}}_{n}$, i.e., $d{z}_{n}/{dt}$,

2 $${{\bf{x}}}_{n}=W{{\bf{z}}}_{n}+{S}_{x}{{\boldsymbol{\varepsilon }}}_{n},$$

where ${{\bf{z}}}_{n}={\left({z}_{n},{{Cz\text{'}}}_{n},1\right)}^{{\rm{T}}}$, $W\in {{\mathbb{R}}}^{L\times 3}$ indicates the weight matrix, ${S}_{x}={\rm diag}({\sigma }_{{x}_{1}},{\sigma }_{{x}_{2}},\ldots ,{\sigma }_{{x}_{L}})\in {R}^{L\times L}$ and ${{\boldsymbol{\varepsilon }}}_{n}\in {{\mathbb{R}}}^{L}$ indicate independent Gaussian noises with zero mean and unit variance, respectively (Fig. 1b, c)$.$ The temporal derivative ${{z\text{'}}}_{n}$ indicates the instantaneous speed of Aβ accumulation derived from the logistic equation. $C$ is a scaling factor common across samples. Here, we assumed that biomarker candidate data ${{\bf{x}}}_{n}$ were generated (Fig. 1b, c) via the same biological process among animals. $W$ is the shared parameter among animals sampled from the same distribution. This model was formulated using a hierarchical Bayesian model (see Methods).

### Few-shot learning procedure to predict Aß accumulation

Based on this model, we aimed to estimate the latent Aβ accumulation ${z}_{n}$ from the observed data on the biomarker candidate ${{\bf{x}}}_{n}$. To this end, we also needed to train the model by estimating the parameters from the data. Here, we made two assumptions, based on the data that were actually available: first, the animals must be sampled once as snapshots, not as a time series, owing to the requirement of euthanization. Second, most samples only included either Aβ accumulation or candidate biomarker data, implying that these were observed in different animal populations and only a small subset of the samples had paired data for the biomarker candidates and Aβ accumulation at the same age. Therefore, there were three types of data: unpaired data on Aβ accumulation, unpaired data on biomarker candidates, and paired data containing both.

Given the above assumptions, we proposed a Bayesian probabilistic approach to train the model by integrating paired and unpaired data via few-shot learning or semi-supervised learning (see Methods). In the first step, using only the unpaired dataset of Aβ accumulation, we pre-trained the model by estimating the distribution of the logistic function parameter $\theta$ for each of the WT and AD model animals (Fig. 1d). Next, using the estimated distributions used in the first step as prior knowledge, we inferred the distribution of all model parameters from the remaining datasets; the unpaired dataset on biomarker candidates was used for unsupervised learning, whereas the paired dataset was used for supervised learning.

After learning the parameters, the model could predict the accumulated level of Aβ, ${z}_{n},$ from the observed data on biomarker candidate ${{\bf {x}}}_{n}$ (Fig. 1d; see Methods).

### Experimental data of AD model mice

**Fig. 2:**
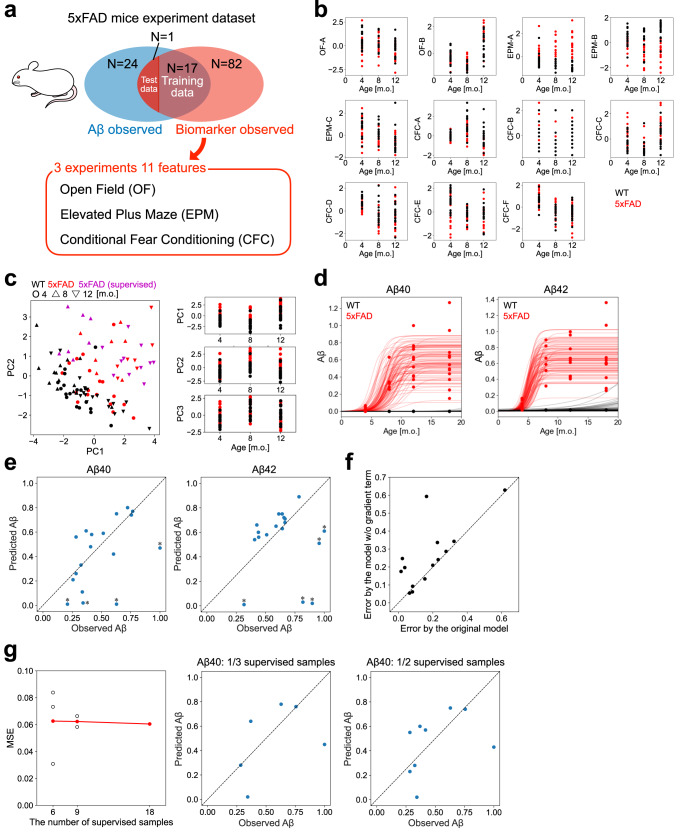
Application of the proposed model to 5xFAD mice data. **a** Components of the experimental data. The accumulation levels of Aβ were observed in 42 samples. The biomarker candidates, 11 features from three different behavioral experiments, were observed in 100 samples. The two groups partially overlapped: 18 samples included Aβ and biomarker candidate data. The paired data samples were all from 5xFAD mice. The predictability was evaluated using leave-one-out cross-validation. **b** Time course of the behavioral features. The black points are from WT mice, and the red points are from 5xFAD mice. **c** PCA of the behavioral features obtained from the experiment. The red and magenta points correspond to 5xFAD samples, and the black points correspond to WT samples. In the scatter plot (left), the point shape represents the sample months for age (circle: 4, triangle: 8, inverted triangle: 12) and the magenta points indicate 5xFAD samples that had the paired data. **d** Inference of hyper-parameters of a logistic function. The data points are scaled insoluble fractions of Aβ_40_ and Aβ_42_ in the hippocampus [no unit]. Red points indicate 5xFAD mice and black points denote WT mice. The lines represent the example logistic functions with hyper-parameters randomly sampled from the learned distribution (red: 5xFAD, black: WT). **e** Accumulation levels of Aβ_40_ and Aβ_42_ at the hippocampus of the samples that had paired data (only for 8- and 12-month-old AD model mice) were predicted by the trained model based on behavioral features. **f** The comparison of the absolute errors by the originally proposed model and the model without the ${z}^{{\prime} }$ term in the hidden state. Samples with Aβ_40_ accumulation levels lower than 0.66 were evaluated. **g** (Left) The mean prediction errors according to the ratio of supervised samples. The black circles represent the results for each number of supervised samples, and the red dots represent the mean for each condition. MSE: mean squared error. (Right) The representative prediction results at the respective conditions.

To apply our estimation method, we adopted real-world experimental data from the AD model and WT mice^22^. The AD model mice were 5xFAD transgenic mice with five human familial AD mutations in APP and PSEN1 on the background of a C57BL/6J strain, showing robust Aβ pathological accumulation and neuronal cell death. Most of the data contained unpaired data either of the Aβ accumulation (24 samples) or behavioral features (82 samples). In contrast, some paired data existed (18 samples, only for 8- and 12-month-old AD model mice; Fig. 2a). Aβ accumulation was evaluated by the insoluble fraction level of Aβ_40_ and Aβ_42_ in the hippocampus of 42 AD model mice at 4, 8, 12, and 18 months of age (Fig. 2d). For WT mice, the observation of Aβ was unavailable in the dataset. We assumed that the insoluble Aβ in WT mice remained undetectable during the mice’s lifetime based on reports that WT mice do not develop Aβ plaque during their normal life span^23^, and prepared virtual samples of the WT mice with the unpaired observation of Aβ accumulation. Each AD model and WT mouse were behaviorally evaluated; we selected three types of behavioral experiments where 11 features were obtained at 4, 8, or 12 months of age (Supplementary Table 1; Fig. 2b). These 11-dimensional data were addressed as biomarker candidate data and visualized using principal component analysis. The distribution of data from the samples that had the paired data was approximately within the range of the distribution of data from the samples that had the unpaired data (Fig. 2c).

### Prediction of Aß accumulation in AD model mice

Using our method, we predicted Aβ accumulation in the hippocampus using behavioral features as biomarker candidates. First, we pre-trained the model to learn the distributions of the parameters of the logistic function based on the insoluble fraction level of Aβ. The pre-trained model generated logistic time courses, representing the observed insoluble fraction level of Aβ_40_ and Aβ_42_ (Fig. 2d). Next, we trained the model using an unpaired dataset of behavioral features and a paired dataset.

Using the trained model, we predicted the accumulation level of Aβ from the behavioral features of the paired data using leave-one-out cross-validation (Fig. 2e). The prediction errors were almost the same for the two types of Aβ (mean squared error [MSE] = 0.060 for Aβ_40_ and MSE = 0.111 for Aβ_42_). Most predictions followed the observed amount of Aβ both in Aβ_40_ and Aβ_42_. However, several data were unpredictable with large errors (asterisks in Fig. 2e). Three of the samples that were not predictable were from 8-month-old mice and shared between Aβ_40_ and Aβ_42_. Moreover, 8-month-old mice showed larger prediction errors than 12-month-old mice (MSE of Aβ_40_ = 0.083 at 8 months, 0.046 at 12 months; MSE of Aβ_42_ = 0.214 at 8 months, 0.046 at 12 months, Supplementary Figure 1a). To identify the cause of the large errors, we predicted the mouse type of the samples based on the behavioral features and found that two of the three samples from 8-month-old mice with large Aβ prediction errors were predicted as WT rather than AD models. All samples that were incorrectly predicted to be from WT mice were from 8-month-old mice. (Supplementary Fig. 1a, b). We then evaluated the importance of the instantaneous speed of Aβ accumulation, ${{z\text{'}}}_{n},$ incorporated at the hidden state, ${{\bf{z}}}_{n},$ of the proposed model. If the term was excluded from the hidden state, the absolute error increased in some samples with observed Aβ accumulation level lower than 0.66 in Aβ_40_ (Fig. 2f). Here, the threshold was set such that all the 8-month-old samples were included below the threshold. The Aβ of the samples may still be in the process of accumulating, suggesting the importance of the term in the very early phase of AD. The accumulation level of Aβ in the cortex was predicted, though the prediction performance was not as good as that in the hippocampus (Supplementary Fig. 2).

We also evaluated the prediction performance when changing the ratio of the paired and unpaired data. We divided the paired data into two equal parts and examined the prediction accuracy when only one of them was used as paired data and the other was used as unpaired data. Similarly, we examined the prediction accuracy of dividing the data into three equal groups, using only one group as paired data and the other as unpaired data. On average, the prediction accuracy did not change much even when only 1/2 or 1/3 of the paired data was used (Fig. 2g).

### Selection of biomarkers to predict Aß accumulation

**Fig. 3:**
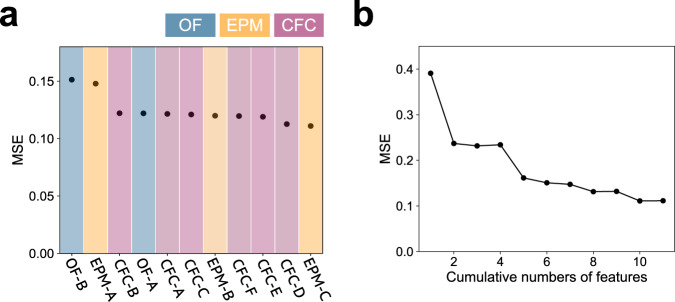
Evaluation of the behavioral features based on the predictability of the Aβ accumulation level. **a** Prediction error in the case of excluding a particular behavioral feature from the observation. **b** Variation of the prediction performance when the number of the observed features increases; the features are sequentially included from the top of the list in Fig. 3a.

To assess the importance of each behavioral feature in predicting Aβ accumulation, we removed each behavioral feature and evaluated the prediction error for each feature (Fig. 3a). We then found that predictive performance was considerably decreased by removing “time in the center” in the open-field experiment and “time spent in the open arm” in the elevated plus maze experiment, which exhibited significant differences between WT and 5xFAD mice^22^. Next, we ranked the features by their impact on the prediction error and assessed the prediction performance by including features individually from the top of the ranks (Fig. 3b). The prediction error decreased considerably until the top five features of the ranks were recruited, which comprised features from three different experiments. The results show that multivariate features from different experiments could be potential AD biomarkers.

We also evaluated the number of features required for the proposed model to determine its predictability. Here, we randomly selected several behavioral features by varying their number, trained the model, and made a prediction (Supplementary Fig. 3a). Statistically significant differences were detected between predictions using 1–7 and 11 features (Mann–Whitney U-test with Holm’s correction for multiple comparisons, *p* < 0.05). In contrast, there was no significant difference between the predictions using 10 and 11 features (Supplementary Fig. 3b), suggesting that as many diverse features as possible are preferable to achieve better prediction performance.

### Prediction of Aß accumulation by conventional machine learning methods

**Fig. 4:**
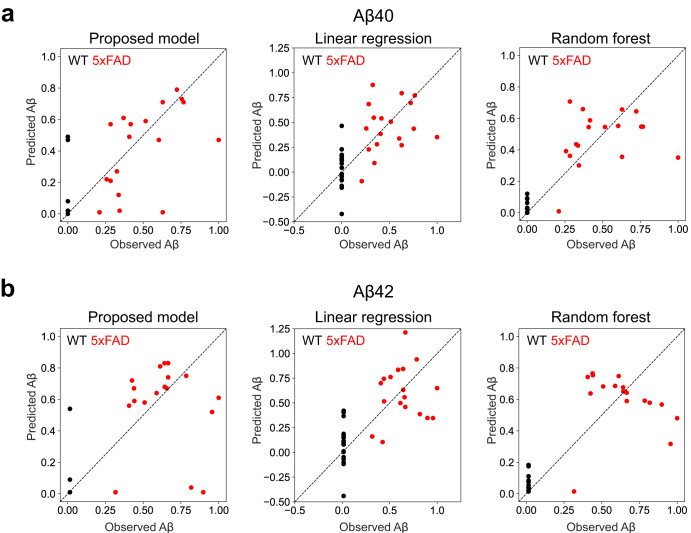
Comparison with standard machine learning methods. Predictive performances against (**a**) Aβ_40_ and (**b**) Aβ_42_ of the proposed model, linear regression, and random forest regression. Eighteen virtually prepared supervised WT samples were used as test samples; the Aβ accumulation levels of such samples were assumed to be the minimum in the observation of Aβ. Three 5xFAD mice and three WT mice were used as test samples and evaluated using 6-fold cross-validation.

The predictive performance of the proposed model was compared with that of conventional machine learning techniques, namely ordinary linear and random forest regressions. To fairly compare prediction performance, we virtually created paired data from randomly selected WT mice and used the mice as a training or test sample for the prediction. We then demonstrated that our proposed model outperformed standard machine learning techniques both in the prediction of Aβ_40_ and Aβ_42_ in the hippocampus (Fig. 4a, b, Supplementary Table 2). The predicted values of Aβ accumulation with the ordinary linear regression overlapped in WT and 5xFAD mice, and the predicted values in some WT mice were negative (Fig. 4a, b). Moreover, the random forest regressor failed to predict the level of accumulation (for instance, large or small) in 5xFAD mice (especially as shown in Fig. 4b**)**. Indeed, the proposed model showed a smaller median absolute prediction error than that of standard models (Supplementary Table 2).

### Application to synthetic data

In the 5xFAD experimental data used in this study, the paired data with Aβ accumulation and behavioral features were limited only to the phase when Aβ accumulation has vastly progressed, i.e., in 8- and 12-month-old mice. Thus, the predictive performance of the proposed model for the earlier phase of Aβ accumulation remains unknown. To evaluate this, the model was applied to synthetic data that contained earlier phase samples.

**Fig. 5:**
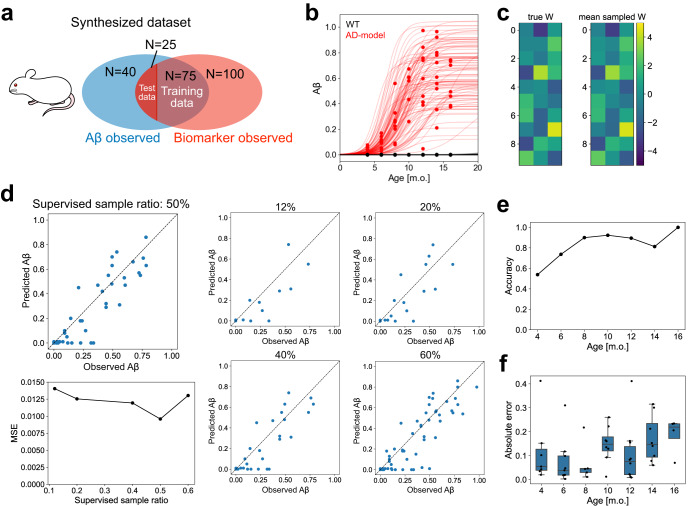
Learning and prediction performances of the proposed model evaluated by synthesized data. **a** Components of the synthesized data. The accumulation levels of Aβ were observed in 140 samples. The biomarker candidates were observed in 200 samples. The 2 groups partially overlap; 100 samples included Aβ and biomarker candidate data. The ages of the samples were randomly sampled from ${t}_{n}=\{4,6,\ldots ,16\}$ and WT and AD model samples were in the same proportion. The predictability was evaluated by 4-fold cross-validation. **b** Inference of hyper-parameters of a logistic function from the observed amount of Aβ. Red points are the observed value of AD model samples; black points correspond to WT samples. The example logistic functions with hyper-parameters were randomly sampled from the learned distribution and are represented by red (AD model) and black (WT) lines. **c** Representative results of the inference of the model parameters from synthetically generated biomarker data. The left is the true generated $W,$ and the right is the mean of the posterior samples of $W$. **d** Prediction of the accumulation level of Aβ from the observed biomarker data by the learned model. The mean prediction errors according to the ratio of supervised samples (bottom left) and the prediction results at the respective conditions (right). **e** Accuracy of predicting the presence or absence of Aβ accumulation according to the age of the samples. **f** Absolute prediction errors in the AD model samples according to the age of the samples. The center lines are medians. The boxes represent the interquartile range, indicating values between the first quartile (25th percentile) and the third quartile (75th percentile). The whiskers extend to show the range of the data, excluding any outliers, which are represented as individual dots.

To this end, we prepared synthetic unpaired and paired data at various ages, including the early phases, by simulating the model. The synthetic data were composed of 20 samples of Aβ accumulation alone, 50 samples of biomarker candidates alone, and 50 samples of paired data for each AD model and WT (Fig. 5a). First, we pre-trained the model using the synthesized unpaired data on Aβ accumulation, representing the variation in the observed accumulation level of Aβ (Fig. 5b). We then trained the model using unpaired data on biomarker candidates and paired data in the manner of few-shot learning. We confirmed that the estimated parameters followed the ground truth used for the synthesized data (Fig. 5c), indicating that our estimation method was efficient. Using the trained model, we predicted ${z}^{* }$, the Aβ accumulation level of unknown samples from the observed biomarker features ${\bf x}^{* }$ (Fig. 5d, top left). We evaluated the prediction accuracy by changing the ratio of supervised paired samples for training. The MSE changed slightly with varying ratios, suggesting that not many supervised samples were required for prediction (right and bottom left in Fig. 5d).

Finally, we evaluated the predictive performance dependency of the samples on age. When assessing whether the true mouse type was consistent with whether the predicted Aβ accumulation levels of zero or non-zero, the presence or absence of Aβ accumulation was predicted relatively accurately in the samples from older mice as compared to those from younger mice, i.e., zero Aβ in the WT mice and non-zero Aβ in the AD model mice (Fig. 5e). The predictive performance of Aβ accumulation in the AD model samples did not change significantly with age (Fig. 5f, Mann–Whitney U-test with Holm’s correction for multiple comparisons). The results suggest that the model can predict Aβ accumulation levels in samples in the early phase of the accumulation process, while it may fail to predict the type of sample in the same phase.

## Discussion

Herein, we proposed a hierarchical Bayesian model that describes how biomarker candidates are generated in response to the accumulation of Aβ in the brain. By integrating mostly unpaired data on Aβ quantification and the behavioral features obtained in behavioral experiments with 5xFAD mice, our model predicted the quantitative accumulation level of Aβ based on behavioral features in most samples. The instantaneous accumulation speed introduced at the hidden state of our model was suggested to play an important role in the prediction, especially during the early phase. Based on the effect of each biomarker candidate on predictability, we revealed that multiple behavioral features from three different behavioral experiments could be important biomarkers for predicting Aβ accumulation level. This study may demonstrate the proof-of-concept of Aβ-predictability-based multivariate AD biomarker discovery.

The proposed model can naturally integrate information from paired and unpaired data. The inference of the distribution of the logistic function parameters from Aβ-observed unpaired data constrains the dynamic range of Aβ accumulation level. The information from unpaired data that lack an observed amount of Aβ provides the generation process of biomarker candidates and their variability. Learning information from the paired supervised data further helps calibrate the generation process. Notably, as the simultaneous observation of biomarker candidates and Aβ from the same sample is labor-intensive, expensive, and technically challenging, most samples usually lack information on either biomarker candidates or Aβ accumulation. In this scenario, the proposed model makes it possible to make such incomplete datasets available for Aβ predictability-based biomarker discovery.

The predictive performance of the proposed model was found to be inferior in young mice than that in older mice. The nature of the analyzed biomarker candidates and the proposed model limitations could have affected the results. The principal component space of the 5xFAD mouse behavioral test data showed little difference between WT and AD model mice in the younger samples (Fig. 2c), which probably caused “misclassification” of some of the 5xFAD mice as WT mice resulting in large prediction errors (Fig. 2e, Supplementary Fig. 1). Such “misclassification” of the two samples was avoided in the prediction by a random forest regressor (Fig. 4a, b). Therefore, the proposed method could be improved by adding a nonlinear process, such as the kernel method, to the process of generating the observed data. We confirmed whether the model with nonlinear transformation yields better prediction performance by adopting a 3-layer neural network at the generation from ${\boldsymbol{z}}$ to ${\bf x}$. However, the prediction performance only improved slightly (data not shown). Since the goal of this study is to contribute to the discovery of biomarkers, we believe that discovering biomarker candidates more suitable for prediction, is also important.

In humans, the accumulation of Aβ may initiate 10–20 years before the recognized cognitive decline^1,2^. However, in model animals, behaviors and cognitive abilities were altered before the saturation of Aβ accumulation. For instance, 5xFAD mice showed a decline in memory function before 6 months of age, when Aβ was still in the accumulation process^22,24,25^. These facts suggest that a probabilistic model sensitive to an earlier stage of Aβ accumulation is preferable to discover biomarkers in model animals. Furthermore, the gradient term of accumulated Aβ at the generation of biomarker data in the model might bestow such specificity to the proposed model when analyzing data that contain phase-specific biomarkers (Fig. 2f).

Whether Aβ acts upstream in the cascade leading to cell death, as in the amyloid hypothesis, is controversial^26,27^. However, Aβ indeed accumulates in the initial stages of AD. Even if there is no causal relationship between the accumulation of Aβ and AD progression, Aβ may be a useful precursor for predicting AD. Notably, studies have reported alterations in the phenotypes of AD model mice that appear earlier than the onset of Aβ accumulation^28^. Such lesions may allow for an earlier definition of the latent state of AD progression.

Machine learning approaches for identifying the latent progression states of AD have recently attracted attention. Probabilistic models, such as mixed effect^29–33^ and hidden Markov models^34–36^, found the latent trajectories of disease progression from longitudinal data of clinical cohorts in unsupervised learning. Our approach differs from those of previous studies in that the proposed model assumed the quantitative accumulation level of Aβ as “the latent progression state” and estimated the state via a few-shot learning approach and directly describes the relationship between Aβ levels and biomarkers. These characteristics should be advantageous in predicting Aβ levels from biomarkers, especially when the number of samples available for training is limited. Furthermore, Aβ may propagate from a brain region to other regions^37,38^, which suggests that the spatial distribution of accumulation could be the hidden state. The molecular biological observation or the direct quantitative observation of Aβ is challenging in humans. Nevertheless, the proposed framework is potentially beneficial for discovering non-invasive convenient biomarkers^8,9,39,40^ that are relevant to the amount of PET-detected Aβ or CSF Aβ in human data.

In other neurodegenerative diseases, such as Parkinson’s disease, Lewy body dementia, multiple system atrophy, Huntington’s disease, amyotrophic lateral sclerosis, and frontotemporal lobar degeneration, abnormal proteins accumulate in specific brain regions, possibly leading to neuronal death^41–43^. The modeling approach presented herein is also potentially applicable to such neurodegenerative diseases. The risk of neurodegenerative diseases is a growing concern in an aging society. Predictability-based biomarker discovery using the proposed model may contribute to identifying biomarkers that make available predictions and potential interventions for diseases.

## Methods

### Generative model of Aß and biomarker candidates

Serial PET scans of humans used for the imaging of Aß^44^ and in vivo imaging of Aß plaques^45^ have demonstrated that the temporal progression of Aß accumulation can be characterized by a sigmoid-shaped trajectory. Based on these previously reported findings, our model assumes that Aß accumulation follows a logistic function:

3 $${z}_{n}=\frac{{\alpha }_{n}}{1+\exp \left(-{\beta }_{n}{t}_{n}+{\gamma }_{n}\right)},$$

where ${\theta }_{n}=\left\{{\alpha }_{n},{\beta }_{n},{\gamma }_{n}\right\}$ is a set of parameters, and $n$ is the animal index. This equation can be rewritten from Eq. (1), where ${\gamma }_{n}={\beta }_{n}{\tau }_{n}$. Accordingly, the temporal derivative of the Aβ accumulation is

4 $${z}_{n}^{{\prime} }={\beta }_{n}{z}_{n}\left(1-\frac{{z}_{n}}{{\alpha }_{n}}\right).$$

The parameter ${\theta }_{n}$ depends on the individual, following distributions as

5 $$P\left({\alpha }_{n}\right)=\prod _{k\in \left\{1,2\right\}}{{\mathscr{N}}}_{+}{\left(\left.{\alpha }_{n}\right|{\mu }_{\alpha ,k},{\sigma }_{\alpha ,k}^{2}\right)}^{{s}_{n,k}},$$

6 $$P\left({\beta }_{n}\right)=\prod _{k\in \left\{1,2\right\}}{{\mathscr{N}}}_{+}{\left(\left.{\beta }_{n}\right|{\mu }_{\beta ,k},{\sigma }_{\beta ,k}^{2}\right)}^{{s}_{n,k}},$$

7 $$P\left({\gamma }_{n}\right)=\prod _{k\in \left\{1,2\right\}}{\mathscr{N}}{\left(\left.{\gamma }_{n}\right|{\mu }_{\gamma ,k},{\sigma }_{\gamma ,k}^{2}\right)}^{{s}_{n,k}},$$

where ${\mathscr{N}}{\mathscr{(}}{x|}\mu ,{\sigma }^{2})$ and ${{\mathscr{N}}}_{+}({x|}\mu ,{\sigma }^{2})$ indicate normal distribution with mean $\mu$ and variance ${\sigma }^{2}$ and truncated normal distribution with a range of $x\, > \,0$, respectively; ${{\bf{s}}}_{n}$ is a one-hot vector representing the type of the samples, i.e., WT as ${\left(\mathrm{1,0}\right)}^{{\rm{T}}}$ or AD model mice as ${\left(\mathrm{0,1}\right)}^{{\rm{T}}}$; ${\mu }_{\phi ,k}$ and ${{\sigma }^{2}}_{\phi ,{k}}$ ($\phi \in \{\alpha ,\beta ,\gamma \}$) indicate parameters of WT ($k=1$) or AD model mice ($k=2$).

The observed amount of Aß, ${y}_{n}$, is generated from ${z}_{n}$ as

8 $$P\left({y}_{n}|{z}_{n}\right)={\mathscr{N}}\left({y}_{n}|{z}_{n},{\sigma }_{y}^{2}\right).$$

The observed data on $L$-dimensional biomarker candidates ${{\bf{x}}}_{n}\in {{\mathbb{R}}}^{L}$ was generated from ${z}_{n}$ and ${z}_{n}^{{\prime} }$ as

9 $$P\left({{\bf{x}}}_{n}|{{\bf{z}}}_{n}\right)={\mathscr{N}}\left({{\bf{x}}}_{n}{\rm{|}}W{{\bf{z}}}_{n},{\Sigma }_{x}\right),$$

where ${{\bf{z}}}_{n}={\left({z}_{n},{C}{{z\text{'}}}_{n},1\right)}^{{\rm{T}}}$, $W\in {{\mathbb{R}}}^{L\times 3}$ indicates the weight matrix, and ${\Sigma }_{x}={\rm diag}({\sigma }_{{x}_{1}}^{2},{\sigma }_{{x}_{2}}^{2},\ldots ,{\sigma }_{{x}_{L}}^{2})$. $C$ indicates a scaling factor common among samples that calibrates the range of ${z\text{'}}$.

### Prior distribution of parameters

For parameter estimation in a Bayesian manner, we introduced the prior distributions of parameters. The hyper-parameters $P\left({\alpha }_{n}\right)$, $P\left({\beta }_{n}\right)$ and $P\left({\gamma }_{n}\right)$ are sampled from the following distributions:

10 $$P\left({\mu }_{\phi ,k}\right){\mathscr{=}}{\mathscr{N}}\left(\left.{\mu }_{\phi ,k}\right|{m}_{\phi ,k},{v}_{\phi ,k}^{2}\right),$$

11 $$P\left({\sigma }_{\phi ,k}^{-2}\right)={\rm{Gamma}}\left(\left.{\sigma }_{\phi ,k}^{-2}\right|{a}_{\phi ,k},{b}_{\phi ,k}\right),$$

where $\phi \in \{\alpha ,\beta ,\tau \}$, ${\rm{Gamma}}\left(\left.x\right|a,{b}\right)$ indicates a Gamma distribution with shape parameter $a$ and rate parameter $b$.

The prior distribution of $W$ was

12 $$P\left({{\bf{w}}}_{l}\right){\mathscr{=}}{\mathscr{N}}\left(\left.{{\bf{w}}}_{l}\right|{\bf{0}},{\sigma }_{{w}_{l}}^{2}{\bf{I}}\right),$$

where ${{\bf{w}}}_{l}$ indicates the $l$-row of the weight matrix $W$. The prior distribution of its hyper-parameter ${\sigma }_{{w}_{l}}^{2}$ was hierarchically introduced as

13 $$P\left({\sigma }_{{W}_{l}}^{-2}\right)={\rm{Gamma}}\left(\left.{\sigma }_{{W}_{l}}^{-2}\right|{a}_{w},{b}_{w}\right).$$

The prior distribution of ${\sigma }_{{x}_{l}}^{2}$ was

14 $$P\left({\sigma }_{{x}_{l}}^{-2}\right)={\rm{Gamma}}\left(\left.{\sigma }_{{x}_{l}}^{-2}\right|{a}_{x},{b}_{x}\right)$$

In the Markov chain Monte Carlo (MCMC) sampling algorithm, we used ${v}_{\alpha ,k}=0.1$, ${a}_{\alpha ,k}=25$, ${b}_{\alpha ,k}=0.5,{v}_{\beta ,k}=0.1$, ${a}_{\beta ,k}=100$, ${b}_{\beta ,k}=1$, ${v}_{\gamma ,k}=1$, ${a}_{\gamma ,k}=10$, ${b}_{\gamma ,k}=1$, ${a}_{w}=100$, ${b}_{w}=1000$, ${a}_{x}=0.5$, ${b}_{x}=1$ and ${\sigma }_{y}=0.05$.

When the model learned the distributions of the hyper-parameters from the Aß observation (step 1), we set ${m}_{\alpha ,k}$, ${m}_{\beta ,k}$ and ${m}_{\gamma ,k}$ as the values estimated by the least square method.

### Bayesian inference of parameters

The model parameters were learned in two steps. In the first step (step 1), we inferred the posterior distributions of the parameters of a logistic function and those of hyper-parameters, given the data on Aβ accumulation as follows:

15 $$\begin{array}{ll}P\left({\theta }_{1:{N}_{y}},{\mu }_{\theta },{\sigma }_{\theta }^{2}\,|{y}_{1:{N}_{y}},{t}_{1:{N}_{y}},{{\bf{s}}}_{1:{N}_{y}}\right) \\ \propto \prod _{n\in {{\mathcal{S}}}_{y}}P\left({y}_{n}|{\theta }_{n}{,{t}_{n},\sigma }_{y}^{2}\right)\prod _{k\in \left\{1,2\right\}}{P\left({\theta }_{n}|{\mu }_{\theta ,k},{\sigma }_{\theta ,k}^{2},{{\bf{s}}}_{n}\right)}^{{s}_{n,k}}P\left({\mu }_{\theta ,k}\right)P\left({\sigma }_{\theta ,k}^{2}\right),\end{array}$$

where ${\mu }_{\theta }=\{{\mu }_{\theta ,1},{\mu }_{\theta ,2}\}$, ${\mu }_{\theta ,k}=\{{\mu }_{\alpha ,k},{\mu }_{\beta ,k},{\mu }_{\gamma ,k}\}$, ${\sigma }_{\theta }^{2}=\{{\sigma }_{\theta ,1}^{2},{\sigma }_{\theta ,2}^{2}\}$, ${\sigma }_{\theta ,k}^{2}=\{{\sigma }_{\alpha ,k}^{2},{\sigma }_{\beta ,k}^{2},{\sigma }_{\gamma ,k}^{2}\}$, and ${{\mathcal{S}}}_{y}$ is the set of samples with Aβ accumulation in the training data, and ${N}_{y}$ is the number of samples in ${{\mathcal{S}}}_{y}$. We assumed that the observed level of Aβ at time $t=0$ in all the samples would be ${y}_{n}=0$. This posterior distribution was estimated using the MCMC sampling algorithms. No-U-Turn samplers (NUTS) were used in this step because it is difficult to derive the closed form of the posterior distribution. From the posterior samples, we estimated the parameters of the Gaussian and Gamma distributions for ${\mu }_{\theta ,k}$ and ${\sigma }_{\theta ,k}^{2}$, respectively; these distributions with the estimated parameters were used as priors for the hyper-parameters. In the first step, MCMC sampling was performed across three independent chains, where 3000 samples were drawn for burn-in, and another 3000 were drawn to estimate the distribution.

In the second step (step 2), we inferred the posterior distributions of all parameters in the model, given the unpaired data on biomarker candidates and the paired data on Aβ accumulation and biomarker candidates. We used the NUTS-within-Gibbs approach for the inference. The weight matrix $W$, the variance of the weight matrix ${S}_{W}=\{{\sigma }_{{W}_{1}}^{2},{\sigma }_{{W}_{2}}^{2},\ldots ,{\sigma }_{{W}_{L}}^{2}\}$, and the variance of the observation noise ${\sigma }_{x}$ were sampled using Gibbs sampling as follows:

The Gibbs sampler for weight matrix $W\!:$

16 $$\begin{array}{ll}P\left(W|{{\bf{x}}}_{1:{N}_{x}},{y}_{1:{N}_{x}},{t}_{1:{N}_{x}},{{\bf{s}}}_{1:{N}_{x}},{\Sigma }_{x},{S}_{W},{\theta }_{1:{N}_{x}},{\mu }_{\theta },{\sigma }_{\theta }^{2}\right)\\ \propto \prod _{n\in {{\mathcal{S}}}_{X}}P\left({{\bf{x}}}_{n}|W,{{\bf{z}}}_{n},{\Sigma }_{x}\right)P\left(W|{S}_{W}\right)=\prod _{l=1}{\mathcal{N}}\left({{\bf{w}}}_{l}|{\widetilde{\mu }}_{{w}_{l}},{\widetilde{\sigma }}_{{w}_{l}}^{2}I\right),\end{array}$$

where ${{\mathcal{S}}}_{X}$ is a set of samples with at least biomarker candidates in the training data, ${N}_{X}$ is the number of samples in ${{\mathcal{S}}}_{X}$, and

17 $${\widetilde{\mu }}_{{w}_{l}}={\widetilde{\sigma }}_{{w}_{l}}^{2}\sum _{n\in {{\mathcal{S}}}_{X}}\frac{{{\bf{z}}}_{n}{x}_{n,l}}{{\sigma }_{{x}_{l}}^{2}},$$

18 $$\frac{1}{{\widetilde{\sigma }}_{{w}_{l}}^{2}}=\sum _{n\in {{\mathcal{S}}}_{X}\,}\frac{{{\bf{z}}}_{n}{{\bf{z}}}_{n}^{{\rm{T}}}}{{\sigma }_{{x}_{l}}^{2}}+\frac{1}{{\sigma }_{{w}_{l}}^{2}}.$$

The Gibbs sampler for observation noise of biomarker candidates ${S}_{x}\!:$

19 $$\begin{array}{ll}P\left({S}_{x}^{-2}|{{\bf{x}}}_{1:{N}_{x}},{y}_{1:{N}_{x}},{t}_{1:{N}_{x}},{{\bf{s}}}_{1:{N}_{x}},W,{S}_{W},{\theta }_{1:{N}_{x}},{\mu }_{\theta },{\sigma }_{\theta }^{2}\right)\\ \propto \prod _{n\in {{\mathcal{S}}}_{X}}P\left({{\bf{x}}}_{n}|W,{{\bf{z}}}_{n},{\Sigma }_{x}\right)\prod _{l=1}P\left({\sigma }_{{x}_{l}}^{-2}\right)=\prod _{l=1}{\rm Gamma}\left({\sigma }_{{x}_{l}}^{-2}|{\widetilde{a}}_{{x}_{l}},{\widetilde{b}}_{{x}_{l}}\right),\end{array}$$

where

20 $${\widetilde{a}}_{{x}_{l}}={a}_{{x}_{l}}+\frac{2}{{N}_{X}},$$

21 $${\widetilde{b}}_{{x}_{l}}={b}_{{x}_{l}}+\sum _{n\in {{\mathcal{S}}}_{X}}\frac{1}{2}{\left({x}_{n,l}-{{\bf{w}}}_{l}^{{\rm{T}}}{{\bf{z}}}_{n}\right)}^{2}.$$

The Gibbs sampler for the variance of the coefficient matrix ${S}_{W}\!:$

22 $$\begin{array}{ll}P\left({S}_{W}^{-1}|{{\bf{x}}}_{1:{N}_{x}},{y}_{1:{N}_{x}},{t}_{1:{N}_{x}},{{\bf{s}}}_{1:{N}_{x}},W,{\Sigma }_{x},{\theta }_{1:{N}_{x}},{\mu }_{\theta },{\sigma }_{\theta }^{2}\right)\\ \propto p\left(W|{S}_{w}\right)\prod _{l=1}P\left({\sigma }_{{w}_{l}}^{-2}\right)=\prod _{l=1}{\rm Gamma}\left({\sigma }_{{W}_{l}}^{-2}|{\widetilde{a}}_{{w}_{l}},{\widetilde{b}}_{{w}_{l}}\right),\end{array}$$

where

23 $${\widetilde{a}}_{{w}_{l}}={a}_{{w}_{l}}+\frac{3}{2},$$

24 $${\widetilde{b}}_{{w}_{l}}={b}_{{w}_{l}}+\frac{1}{2}{{\bf{w}}}_{l}^{T}{{\bf{w}}}_{l}.$$

The posterior distribution of other parameters was sampled by MCMC using NUTS as

25 $$\begin{array}{ll}P\left({\theta }_{1:{N}_{{pair}}},{\mu }_{\theta },{\sigma }_{\theta }^{2}\,|{{\bf{x}}}_{1:{N}_{x}},{z}_{1:{N}_{x}}^{o},{t}_{1:{N}_{x}},{{\bf{s}}}_{1:{N}_{x}},W,{\Sigma }_{x},{S}_{W}\right)\\=P\left({\mu }_{\theta }\right)P({\sigma }_{\theta }^{2})\left[\prod _{n\in {{\mathcal{S}}}_{{XY}}}P\left(\right.{{\bf{x}}}_{n}\left({{\bf{z}}}_{n},W,{\Sigma }_{x}\right)P\left({y}_{n}|{{\bf{z}}}_{n},{\sigma }_{z}^{2}\right)P\left({\theta }_{n}|{\mu }_{\theta },{\sigma }_{\theta }^{2},{{\bf{s}}}_{n}\right)\right]\\ \qquad\times \begin{array}{c}\left[\prod _{{n}^{{\prime} }\in {{\mathcal{S}}}_{X}}P\left(\right.{{\bf{x}}}_{{n}^{{\prime} }}\left({{\bf{z}}}_{{n}^{{\prime} }},W,{\Sigma }_{x}\right)P\left({\theta }_{{n}^{{\prime} }}|{\mu }_{\theta },{\sigma }_{\theta }^{2},{{\bf{s}}}_{{n}^{{\prime} }}\right)\right],\end{array}\end{array}$$

where ${{\mathcal{S}}}_{{XY}}$ and ${{\mathcal{S}}}_{X}$ are sets of samples with paired data on Aβ accumulation and biomarker candidates and unpaired data on biomarker candidates, respectively. We adopted the distributions estimated in Step 1 as $P\left({\mu }_{\theta }\right)$ and $P({\sigma }_{\theta }^{2})$. In the second step, MCMC sampling was performed across three independent chains, where 5000 samples were drawn for burn-in, and another 5000 were drawn to estimate the distribution. The inference program was implemented in Python using the NumPyro framework.

### Prediction of Aß accumulation from biomarkers

In the prediction of Aß accumulation in the test data (step 3), we computed a conditional posterior predictive distribution of ${y}^{* }$ given ${{\bf{x}}}^{* }$ using the following equation:

26 $$P\,\left({y}^{* }|{{\bf{x}}}^{* },D\right)\propto P\,\left({{\bf{x}}}^{* },{y}^{* }|\,D\right)\propto \frac{1}{2{N}_{t}I}\mathop{\sum}\limits_{i=1}\mathop{\sum}\limits_{t}\mathop{\sum}\limits_{\bf s}P\,\left({\bf{x}}^{* }|t,{\bf s},{W}^{\left(i\right)},{\Sigma }_{x}^{\left(i\right)},{{\rm{S}}}_{W}^{\left(i\right)},{\theta}^{\left(i\right)}\right)P\,\left({y}^{* }|t,{\bf s},{\theta }^{\left(i\right)}\right),$$

where $D$ is the learned training data, ${W}^{\left(i\right)}$, ${\Sigma }_{x}^{(i)}$, ${{\rm{S}}}_{W}^{(i)}$, and ${\theta }^{(i)}$ are the posterior samples of the parameters, $I$ is the number of posterior samples, and ${N}_{t}$ is the number of time points considered in the prediction $t=\{2,3,\ldots ,18\}$.

Similarly, in the mouse type prediction in the test data, we computed a conditional posterior predictive distribution of ${\bf s}^{* }$ given ${{\bf{x}}}^{* }$ using the following equation:

27 $$\begin{array}{ll}P\,\left({\bf s}^{* }|{{\bf{x}}}^{* },D\right)\propto P\,\left({{\bf{x}}}^{* },{\bf s}^{* }|\,D\right)\\ \propto \frac{1}{2{N}_{t}I}\mathop{\sum}\limits_{i=1}\mathop{\sum}\limits _{t}P\,\left({{\bf{x}}}^{* }|t,{\bf s}^{* },{W}^{\left(i\right)},{\Sigma }_{x}^{\left(i\right)},{{\rm{S}}}_{W}^{\left(i\right)},{\theta }^{\left(i\right)}\right).\end{array}$$

### Behavioral experiments with 5xFAD mice

We used the dataset previously described in Forner et al.^22^, obtained from a public repository (AD Knowledge Portal; https://adknowledgeportal.synapse.org/). Eleven features from three experiments were analyzed using our proposed model. In the open-field experiment, the velocity and time ratio in the center (the time in the center divided by the time in the arena) was used in the analysis. In the elevated plus maze experiment, the amount of time a mouse spent cumulatively in the open arm, closed arm, and center area of the maze was used in the analysis. In the contextual fear conditioning experiment, the activity level, inactive freezing frequency, and cumulative duration of inactive freezing were monitored for each mouse during 2-min habituation and exploration in a chamber. Subsequently, an electrical shock was applied to the mouse. After 24 h, the same behavioral features were monitored for 5 min in the chamber.

### Preprocessing

Preprocessing was performed to analyze 5xFAD mouse behavioral data. Behavioral features were standardized such that the mean and standard deviation of each feature were 0 and 1.0, respectively. The observed amount of Aβ was scaled so that the maximum observed value for 12-month-old mice equaled 1.0. Based on the assumption that insoluble Aβ in the brain of WT mice remains undetectable throughout their lives, we virtually generated unpaired-Aβ-observation WT samples at 8, 12, and 18 months of age, where the observed amount of Aβ at each time sample was 0.0. A 5xFAD mouse “individual ID = 572” was excluded from the paired-data samples because the measurement of insoluble Aβ in the sample may have failed.

### Comparison with linear regression and a random forest regressor

To fairly compare the prediction performance of the proposed method and conventional machine learning methods against both WT and 5xFAD mice, we prepared supervised samples of the WT mice and 5xFAD mice. We randomly selected 18 WT mice, all of which had unpaired behavioral data, and provided paired data in which the Aβ level was observed to be zero using them as samples with the paired data. The standard machine learning methods were implemented using the scikit-learn module in Python. The number of trees in the random forest regressor was set to 100.

### Reporting summary

Further information on research design is available in the Nature Research Reporting Summary linked to this article.

### Supplementary information

Supplemental Information

Reporting Summary

## Data Availability

Datasets for the current study are obtained from the AD Knowledge Portal (https://adknowledgeportal.synapse.org/).

## Code availability

All code used in the current study is available on the GitHub repository. (https://github.com/yadapo/PredictionAbeta).
