# Supplementary material for: Few-shot prediction of amyloid β accumulation from mainly unpaired data on biomarker candidates
Source: NPJ Syst Biol Appl. 2023 Nov 23;9:59. doi: 10.1038/s41540-023-00321-5 (PMC10665362; doi:10.1038/s41540-023-00321-5)
Supplement: Supplementary file 1 — Supplemental Information [file 41540_2023_321_MOESM1_ESM.pdf]

Supplementary Figure 1

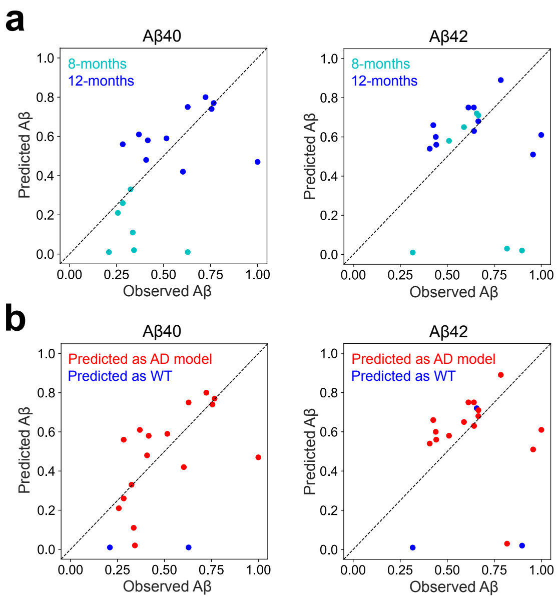

Supplementary Figure 1: Aß level prediction results based on sample age and predicted mouse type.

The results show differences in the quantitative prediction of $Aß_{40}$ and $Aß_{42}$ accumulation in the hippocampus by sample age in months (**a**) and by predicted mouse type (**b**). (**a**) The cyan points indicate samples at 8 months of age and the blue points indicate samples at 12 months of age. (**b**) The red points are samples correctly predicted to be AD model mice; the blue points are samples incorrectly predicted to be WT mice.

Supplementary Figure 2

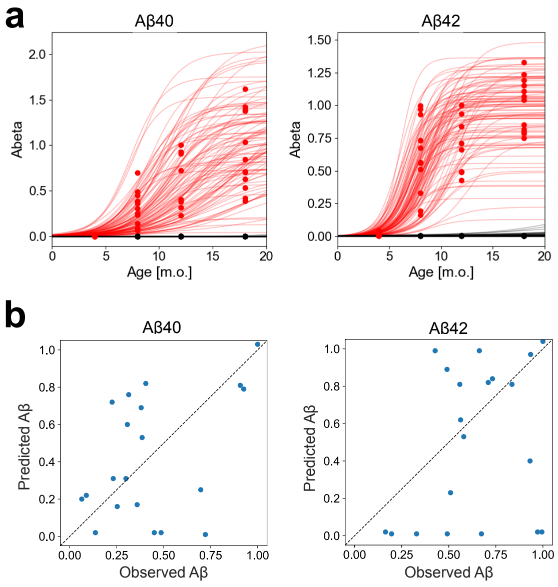

Supplementary Figure 2: Results of the learning and prediction of Aß in the cortex.

(**a**) The inference of hyper-parameters of a logistic function. The red points indicate AD model samples and the black points denote WT samples. The lines represent the example logistic functions whose hyper-parameters were randomly sampled from the learned distribution (red: 5xFAD, black: WT). (**b**) The results in the quantitative predictions of accumulation of $Aß_{40}$ and $Aß_{42}$ at the cortex by the trained model.

Supplementary Figure 3

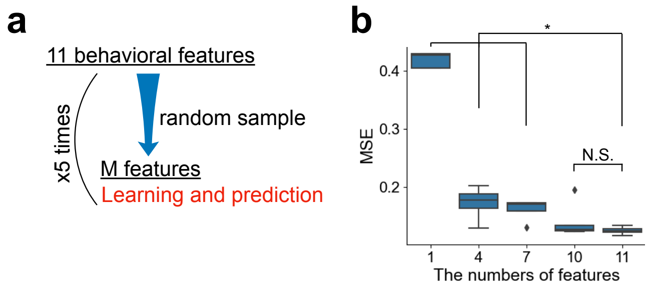

Supplementary Figure 3: Learnings and predictions in randomly sampled features.

(**a**) M features are randomly sampled from 11 behavioral features; the model learns the data using the M features, and the prediction performance is evaluated. (**b**) Mean squared errors between the observed amount of Aβ ($A\beta_{42}$ at the hippocampus) and the predicted amount using $M = \{1, 4, 7, 10, 11\}$ features for the learning and prediction. M features were sampled five times. The center lines are medians. The boxes represent the interquartile range, indicating values between the first quartile (25th percentile) and the third quartile (75th percentile). The whiskers extend to show the range of the data, excluding any outliers, which are represented as individual dots. *$p<0.05$. Mann–Whitney U-test with Holm's correction for multiple comparisons.

Supplementary Table 1: Behavioral experiments and features from Forner et al. used in this study.

Behavioral experiment	Index	Feature
Open field (OF)	A	Velocity
	B	Time ratio in the center*
Elevated plus maze (EPM)	A	Cumulative open arms
	B	Cumulative closed arms
	C	Cumulative center
Conditional fear conditioning (CFC)	A	Baseline train activity within arena mean
	B	Baseline train inactive freezing frequency
	C	Baseline train inactive freezing cumulative duration
	D	test activity within arena mean
	E	test inactive freezing frequency
	F	test inactive freezing cumulative duration

*defined as “time in the center” / “time in the arena”.

Supplementary Table 2: Comparison of predictive performance with standard machine learning techniques.

Method	Aß	Sample type	Median absolute prediction error
Proposed	$Aß_{40}$	5xFAD	0.108
		WT	0.0
	$Aß_{42}$	5xFAD	0.177
		WT	0.005
Linear regression (LR)	$Aß_{40}$	5xFAD	0.197
		WT	0.133
	$Aß_{42}$	5xFAD	0.249
		WT	0.125
Random forest (RF)	$Aß_{40}$	5xFAD	0.136
		WT	0.003
	$Aß_{42}$	5xFAD	0.208
		WT	0.005
